# Sildenafil Citrate Influences Production of TNF-*α* in Healthy Men Lymphocytes

**DOI:** 10.1155/2019/8478750

**Published:** 2019-10-22

**Authors:** Michał Zych, Aleksander Roszczyk, Monika Kniotek, Beata Kaleta, Radoslaw Zagozdzon

**Affiliations:** ^1^Department of Clinical Immunology, Transplantation Institute, Medical University of Warsaw, Nowogrodzka 59, 02-006 Warsaw, Poland; ^2^Department of Immunology, Transplantology, and Internal Diseases, Medical University of Warsaw, Nowogrodzka 59, 02-006 Warsaw, Poland

## Abstract

The aim of our study was to determine whether sildenafil citrate influences the production of Th1- (TNF-*α*, INF-*γ*) or Th2-type (TGF-*β*, IL-10) cytokines by lymphocytes of healthy men. Sildenafil citrate (SC) is a selective blocker of phosphodiesterase 5, by competing for the binding site with cGMP. It was reported that a higher risk of sexually transmitted diseases (STD) could be correlated with a recreational use of sildenafil, especially when combined with another drug. While behavioral causes of these findings are understood, it is worth considering other causes of that phenomenon that might rely on the influence of sildenafil on the immune system. *Material and Methods*. Peripheral blood mononuclear cells (PBMCs) were isolated from 27 healthy men donors and cultured in the presence of SC at a concentration of 400 ng/ml. The first set of research was performed on cells stimulated, for at least 4 hours, by incubation with phorbol myristate acetate (PMA), ionomycin, and Golgi-Stop. Subsequently, we determined cytokine production in cells stimulated with phytohemagglutinin (PHA) for 12 hours in the presence of Golgi-Stop. Flow cytometry immunophenotyping of PBMC was performed towards the surface marker of T cells: CD3 and intracellular cytokine expression: TNF-*α*, IFN-*γ*, TGF-*β*, and IL-10. Our findings show that SC significantly decreased the percentage of T cells producing TNF-*α* and displayed tendency to decrease IFN-*γ*, when stimulated with PMA. Frequent usage of SC might strengthen this effect. That could partially explain the impaired immune response to the pathogens of men using the drug.

## 1. Introduction

Phosphodiesterase type 5 (PDE5) is cytosolic phosphohydrolase, belonging to the PDE family, that selectively hydrolyzes cGMP to GMP and cAMP to AMP. The affinity of PDE5 to the cGMP is 100-fold higher than to cAMP [1]. PDE5 is widely distributed among various cell types, for instance, airway smooth muscle cells, vascular smooth muscle cells, epithelial cells, and fibroblasts as well as platelets and monocytes [2]. The majority of PDEs in lymphocytes are PDE3 and PDE4; yet, low activities of PDE1, PDE2, and PDE5 were also found [3]. Cyclic GMP and cAMP are secondary messengers in the intracellular signal transduction. Thus, blockers of phosphodiesterases might change specific signaling pathways [4].

Sildenafil is known as a selective inhibitor of PDE5 [4]. However, it was shown that sildenafil is also able to inhibit PDE1-4 and PDE6 [5]. Inhibition of PDE5 results in an increased concentration of cellular cGMP, which interact with protein kinase G (PKG) leading to activation of ion channel conductance, cellular apoptosis, and glycogenolysis [6, 7].

The ability of sildenafil to induce vascular smooth muscle relaxation and vasodilatation is widely utilized in the treatment of pulmonary hypertension and erectile dysfunction [5, 8]. Few studies have focused on the potential usage of this drug in oncology and immunology. For instance, Serafini et al. showed in their work that sildenafil reduces tumor-induced immunosuppressive mechanism in several tumor types in mice [9].

Tenor et al., in turn, provided evidence of phosphodiesterases 1-5 presence in CD4^+^ and CD8^+^ lymphocytes, with predominant activity of PDE3 and PDE4 [3]. It is then of interest to explore the potential actions of PDE inhibitors of T lymphocyte functions, e.g., cytokine secretion [10, 11]. Cytokines secreted by lymphocytes are regulatory factors in the immune system and play an important role in immunological response [12–14]. Th1 cells produce mostly proinflammatory cytokines such as tumor necrosis factor alpha (TNF-*α*), interferon gamma (IFN-*γ*), and interleukin 2 (IL-2) which mediate cellular immune responses to pathogens, whereas Th2 cells produce mainly interleukin 10 (IL-10), interleukin 5 (IL-5), interleukin 4 (IL-4), and interleukin 13 (IL-13) [15].

As sildenafil is applied in erectile dysfunction treatment, but also used recreationally, the knowledge on the effects of this drug on the immune system of healthy men is of importance. Therefore, the aim of our study was to determine if sildenafil citrate influences the production of particular Th1 (TNF-*α*, INF-*γ*) and Th2 (TGF-*β*, IL-10) cytokines by lymphocytes of healthy men.

## 2. Materials and Methods

The study was approved by the Bioethics Committee of the Medical University of Warsaw. All measurements, interventions, and blood collections were performed after informed consent.

### 2.1. Healthy Blood Donors

Blood samples were obtained from 27 healthy men volunteers, age between 18 and 45, from the Regional Center for Blood Donation and Blood Treatment in the Clinical Hospital of the Infant Jesus University Centre of Science in Warsaw.

### 2.2. Cell Cultures

Blood was vested in heparin tubes. PBMCs from healthy human volunteers were isolated by Histopaque-1077 (Sigma-Aldrich, Germany) gradient centrifugation. The cells from the interphase were harvested, washed in 0.9% NaCl (Kabe, Germany), and suspended at a density of 1 × 10^6^ cells/ml in RPMI supplemented with 10% heat-inactivated fetal calf serum (Sigma-Aldrich) 2 mM glutamine (Sigma-Aldrich, Germany), Antibiotic-Antimycotic solution 100 I.U. penicillin, 100 *μ*g/ml streptomycin, and 0.25 *μ*g/ml amphotericin (Corning, BD, USA). Cells were divided into 2 groups with or without addition of 400 ng/ml SC (Sigma-Aldrich) and cultured in 24-well plates (Nunc, Thermo Fisher, USA), then incubated for 48 hours at 37°C in 5% CO_2_ atmosphere. Initially, research was performed on cells stimulated for 4 hours by incubation with 50 ng/ml PMA (Sigma-Aldrich), 1 *μ*g/ml ionomycin (Sigma-Aldrich), and 4 *μ*l/ml Golgi-Stop (Becton Dickinson (BD), USA). Subsequently, we determined cytokine production in cells stimulated with 2 *μ*g/ml PHA (Sigma-Aldrich) for 12 hours in the presence of 4 *μ*l Golgi-Stop (Becton Dickinson, USA) per 1 ml of culture.

### 2.3. Flow Cytometry Staining and Permeabilization

Cultured cells were washed by centrifugation in Stain Buffer (BD Pharmingen). Cells were suspended in 100 *μ*l Stain Buffer and stained with monoclonal antibody specific for a given cell surface antigen: CD3-PerCP (SK7-clone, Becton Dickinson, USA), CD4-APC-Cy7 (SK3 clone, Becton Dickinson, USA), and CD8-APC (SK1 clone, Becton Dickinson, USA) for 15 min in room temperature in the dark. After that, cells were washed two times in 1 ml of Stain Buffer (Becton Dickinson, Pharmingen, USA), 2000 rpm, 5 minutes. Next, cells were permeabilized with 300 *μ*l BD Cytofix/Cytoperm solution (BD, USA) for 20 minutes in 4°C. After that, cells were washed two times in 1 ml BD Perm/Wash™ buffer 2000 rpm, 5 minutes. After washing, cells were suspended in 100 *μ*l Perm/Wash solution and stained with cytokine-specific antibodies: anti-IFN-*γ*-PE-Cy7 (B27 clone, Becton Dickinson), anti-TNF-*α*-FITC (MAb11 clone, Becton Dickinson), anti-IL-10-PE (JES3-9D7 clone, Becton Dickinson), and anti-TGF-*β*–PE (TW4-9E7 clone, Becton Dickinson) for 30 minutes in 4°C. After intracellular staining, cells were washed once in 1 ml Perm/Wash (Becton Dickinson, USA) 2000 rpm, 5 minutes. After the final wash, cells were resuspended in 300 *μ*l Stain Buffer. Gating strategy and cut-off values of positive fluorescence were based on fluorescence minus one (FMO) experiments and are shown in the supplementary data. Cell readouts were acquired using a Becton Dickinson FACSCanto II cytometer (BD FACSCanto II, Becton Dickinson, USA) and were analyzed with BD FACS Diva 6.1.3. software. Analyzes were conducted on live cells.

### 2.4. Statistical Analysis

Gathered data distribution was tested for normality with the Shapiro-Wilk test. The Wilcoxon paired test was used for those series where difference between series did not have normal distribution; the Student *t*-test was performed for those with normal distribution. The data was statistically analyzed with Statistica 13.1; figures were made in GraphPad Prism 7.

## 3. Results

**Figure 1:**
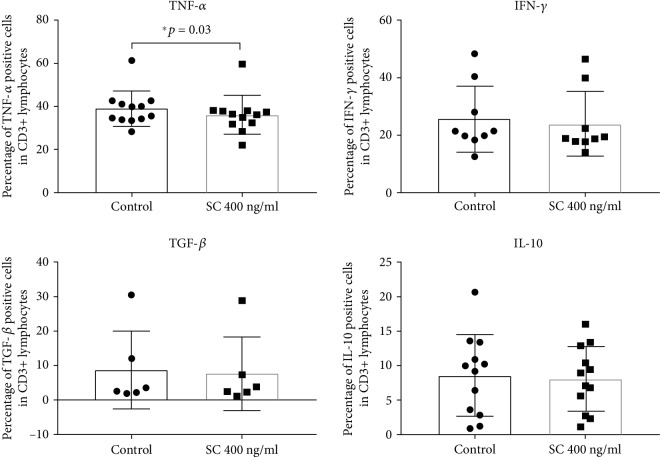
Effect of sildenafil citrate on the percentage of PMA-stimulated T lymphocytes producing cytokines. The error bars represent the standard deviation. Abbreviations: control: PBMC, SC 400 ng/ml: PBMC+SC, ^∗^*p* < 0.05, PBMC: peripheral blood mononuclear cells, SC: sildenafil citrate (TNF-*αn* = 12, IFN-*γn* = 9, TGF-*βn* = 6, and IL-10 *n* = 12); please refer to the supplementary information for gating strategy.

The pilot study performed on PBMCs stimulated with PMA and ionomycin showed that after incubation with sildenafil citrate, the percentage of TNF*α*-producing T lymphocytes was significantly decreased (*p* < 0.05), while other studied cytokine-producing cell percentages were not statistically different in the presence of sildenafil citrate comparing to culture without the drug. However, incubation with SC demonstrated a trend to decrease the percentage of cells expressing each of the studied cytokine (Figure 1).

**Figure 2:**
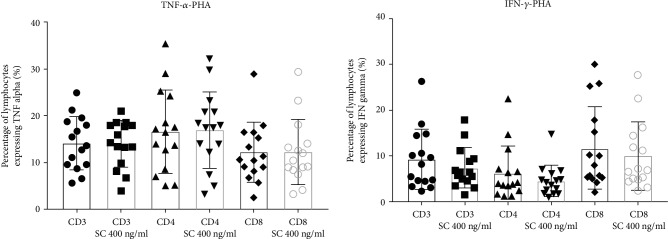
Effect of sildenafil citrate on the percentage of TNF-*α*- and IFN-*γ*-positive T cell subsets after PHA stimulation of PBMC cultures. The error bars represent the standard deviation. Abbreviations: CD3, CD4, and CD8—PBMC, CD3 SC 400 ng/ml, CD4 SC 400 ng/ml, and CD8 SC 400 ng/ml—PBMC+SC, PHA: phytohemagglutinin at a concentration of 2 *μ*g/ml, PBMC: peripheral blood mononuclear cells, SC: sildenafil citrate (*n* = 15); please refer to the supplementary information for gating strategy.

In our extended study, we performed similar research with PBMC after stimulation with PHA. There was no statistically significant difference in the percentage of any studied cytokine-producing cell population in culture with sildenafil citrate comparing to cells incubated without the drug (Figure 2).

## 4. Discussion

It has been observed that the recreational use of sildenafil is associated with a higher risk of sexually transmitted diseases (STD) and it is considered as a risk factor for STD [16], especially when combined with other drugs [17]. While behavioral causes of these findings are understood, it is worth considering other causes of that phenomenon that might rely on the influence of sildenafil on the immune system. In this study, we have observed a significant decrease in the percentage of cells producing TNF-*α* after stimulation with PMA and ionomycin. TNF-*α* plays an important role in inflammation process inter alia as leukocyte activator, inductor of lymphocyte proliferation, and promotor of adhesion and migration of leukocytes [18]. Moreover, patients treated with TNF alpha inhibitors are more likely to develop infectious diseases [19, 20].

Result of current experiment corresponds to our previous findings of downregulating NK cells activity by SC [8]. Kaleta and Boguska reported that SC did not influence T cell proliferation and had no cytotoxic effect on T cells which is in line with our observations [21]. Researchers in opposite to our findings show that cultures of lymphocytes of healthy men treated with sildenafil citrate have an increased level of TNF-*α* in supernatants collected from PBMC culture [22]. The differences in outcomes could be explained with the differences in methods which have been used in research. Specifically, in the current study, we measured TNF-*α* intracellularly only in T lymphocytes, while Kaleta and Boguska used the ELISA test to determine TNF-*α* concentration in culture supernatants from PBMC cultures. Notably, PBMC isolated by gradient centrifugation contain monocytes that are able to secrete large amounts of TNF-*α* after stimulation which have not been our point of interest.

Our results obtained on human lymphocytes were comparable to results obtained on mouse lymphocytes in animal model studies. It has been shown that PDE inhibitors can modulate Th1/Th2/Treg cytokine production. Szczypka and Obminska-Mrukowicz showed that accumulation of cGMP caused by sildenafil citrate reduced the production of proinflammatory cytokine IL-2 in healthy mice [10, 23]. In our studies, we observed similar trend to reduce the production of proinflammatory cytokine, but in this case, SC reduced the production of TNF-*α*. In research presented by Karakhanova et al. [24], sildenafil citrate did not influence the serum concentration of IL-10 in healthy mice watered with the drug. Interestingly, researchers observed a gender-specific effect of the drug [24]. Likewise, in our studies, we did not observe influence of SC for IL-10 production. Karakoyun et al. investigated the effect of sildenafil treatment on rats with colitis. Comparable to our results, sildenafil citrate decreased the level of TNF-*α* and did not affect the level of IL-10 [25]. Kosutova et al. found that sildenafil citrate reduced the level of TNF-*α* and IL-6 in supernatants obtained from homogenized lung tissue rabbits with acute lung injury [26]. Research conducted by Nunes et al. on the mouse model of multiple sclerosis demonstrated a strong anti-inflammatory effect of SC, i.e., sildenafil citrate decreased the level of TNF-*α*, IFN-*α*, IL-2, and IL-1*β* in serum [27]. Nunes et al. reported that sildenafil citrate was managed to reduce the level of IFN-*α*, but only in case when sildenafil citrate was administrated in bolus [27]. Results obtained by Nunes et al. on mouse models are comparable to our findings performed on human lymphocytes considering the production of TNF-*α*. Pifarre et al. observed that sildenafil significantly reduced IFN-*γ* release. The effect was stronger than observed in our studies. The tendency to decrease the level of TNF-*α* after sildenafil oral administration was also confirmed. Researchers did not observe the effect of SC on IL-10 production which is in line with our results [28]. Interestingly, studies performed by Guimaraes et al. showed that treatment with sildenafil was associated with decreased vascular TGF-*β* level in renovascular hypertensive rats which confirm the tendency that we observed in our research [29].

Our current observations have shown that sildenafil citrate had a significant effect on lymphocyte cultures treated with PMA and had no effect on cultures treated with PHA. It is well described that PMA activation is mediated directly by protein kinase C (PKC) [30], while the PHA pathway is associated with the CD2 lectin activation pathway [31, 32] and nuclear factor of activated T cells (NF-AT) [33]. This observation suggests pathway-specific effects of sildenafil citrate via cGMP accumulation on PKC and mitogen-activated protein kinase (MAPK) activation pathway. Zhao et al. established that sildenafil blocked the phosphorylation and degradation of I*κ*B*α* in the NF-*κ*B activation pathway and inhibited the phosphorylation of MAPK, extracellular signal-regulated kinases 1 and 2 (ERK1/2), p38 MAPK, and c-Jun N-terminal kinase (JNK) [34]. Inhibition of NF-*κ*B signaling leads to impaired production of IL-1, IL-6, and TNF-*α*. Inactivation of MAPK could lead to restrain of interferon production.

In summary, our investigation shows that sildenafil citrate administrated in vitro to PBMC derived from healthy men can influence the immune system by lowering TNF production from T lymphocytes. That can be classified as anti-inflammatory and immunosuppressive action; however, more research in this area is needed, including in vivo studies, to determine the significance of this phenomenon.

## 5. Conclusions

In this study, sildenafil citrate decreased significantly the percentage of T cells producing TNF-*α* and displayed a trend to decrease IFN-*γ* as well. While influence of sildenafil on T lymphocytes in our study appears marginal, it is worth noticing that we investigated difference in cytokine production after a single dosage of drug. Frequent usage of sildenafil citrate might strengthen this effect. That could explain the impaired immune response to the pathogens of men using the drug.

## Data Availability

The Sildenafil.xlsx data used to support the findings of this study are available from the corresponding author upon request.

## Abbreviations

AMP: Adenosine monophosphate
cAMP: Cyclic adenosine monophosphate
cGMP: Cyclic guanosine monophosphate
ERK1/2: Extracellular signal-regulated kinases 1 and 2
GMP: Guanosine monophosphate
IL: Interleukin
JNK: c-Jun N-terminal kinase
MAPK: Mitogen-activated protein kinase
NF-AT: Nuclear factor of activated T cells
NF-*κ*B: Nuclear factor kappa-light-chain-enhancer of activated B cells
NK cells: Natural killer cells
NO: Nitric oxide
NOS: Nitric oxide synthase
PBMCs: Peripheral blood mononuclear cells
PBS: Phosphate buffered saline
PDE: Phosphodiesterase
PHA: Phytohemagglutinin
PKC: Protein kinase C
PKG: Protein kinase G
PMA: Phorbol myristate acetate
SC: Sildenafil citrate
STD: Sex transmitted disease
TGF-*β*: Transforming growth factor-*β*
TNF-*α*: Tumor necrosis factor-*α*.

## Supplementary Materials

Supplementary Materials Supplementary Data to Figure 1: lymphocytes after PMA stimulation. Gating strategy: lymphocytes were identified on FSC/SSC dot plot (G1) as cells within 50‐100 × 10^3^ on FSC and below 50 × 10^3^ on SSC. (G2) Doublets exclusion. (G3) CD3-positive cells (T lymphocytes were identified as events with PreCP fluorescence above 10^3^ on logarithmic scale). (G4) TNF-*α*-positive T lymphocytes were identified as CD3^+^ lymphocytes from G3 gate with fluorescence higher than fluorescence minus one from G4a. (G5) IL-10-positive T lymphocytes were identified as CD3^+^ lymphocytes from G3 gate with fluorescence higher than fluorescence minus one from G5a. (G6) INF-*γ*-positive T lymphocytes were identified as CD3^+^ lymphocytes from G3 gate with fluorescence higher than fluorescence minus one from G6a. (G7) TGF-*β*-positive T lymphocytes were identified as CD3^+^ lymphocytes from G3 gate with fluorescence higher than fluorescence minus one from G7a. FMO controls are represented on G4a, G5a, G6a, Ga7, and G8a dot plots. Supplementary Data to Figure 2: lymphocytes after PHA stimulation. Gating strategy: lymphocytes were identified on FSC/SSC dot plot (S1) as cells within 50‐100 × 10^3^ on FSC and below 50 × 10^3^ on SSC, (S2) CD3-positive cells (T lymphocytes were identified as events with PreCP fluorescence above 10^3^ on logarithmic scale). (S3) CD4-positive cells (T helper lymphocytes were identified as events with APCCy7 fluorescence above 10^2^ on logarithmic scale) and CD8-positive cells (T cytotoxic lymphocytes were identified as events with APC fluorescence above 10^3^ on logarithmic scale). IFN-*γ*^pos^ cells were identified as cells positive for PeCy7 above 10^3^, respectively, from gate CD3 (S4a), CD4 (S5a), and CD8 (S6a). TNF-*α*^pos^ cells were identified as cells positive for PeCy7 above 10^3^, respectively, from gate CD3 (S7a), CD4 (S8a), and CD8 (S9a). FMO controls are represented on S4, S5, S6, S7, and S8 histograms.
