# Supplementary material for: Sildenafil Citrate Influences Production of TNF-α in Healthy Men Lymphocytes
Source: J Immunol Res. 2019 Oct 22;2019:8478750. doi: 10.1155/2019/8478750 (PMC6930755; doi:10.1155/2019/8478750)
Supplement: Supplementary Materials — Supplementary Data to Figure 1: lymphocytes after PMA stimulation. Gating strategy: lymphocytes were identified on FSC/SSC dot plot (G1) as cells within 50‐100 × 103 on FSC and below 50 × 103 on SSC. (G2) Doublets exclusion. (G3) CD3-positive cells (T lymphocytes were identified as events with PreCP fluorescence above 103 on logarithmic scale). (G4) TNF-α-positive T lymphocytes were identified as CD3+ lymphocytes from G3 gate with fluorescence higher than fluorescence minus one from G4a. (G5) IL-10-positive T lymphocytes were identified as CD3+ lymphocytes from G3 gate with fluorescence higher than fluorescence minus one from G5a. (G6) INF-γ-positive T lymphocytes were identified as CD3+ lymphocytes from G3 gate with fluorescence higher than fluorescence minus one from G6a. (G7) TGF-β-positive T lymphocytes were identified as CD3+ lymphocytes from G3 gate with fluorescence higher than fluorescence minus one from G7a. FMO controls are represented on G4a, G5a, G6a, Ga7, and G8a dot plots. Supplementary Data to Figure 2: lymphocytes after PHA stimulation. Gating strategy: lymphocytes were identified on FSC/SSC dot plot (S1) as cells within 50‐100 × 103 on FSC and below 50 × 103 on SSC, (S2) CD3-positive cells (T lymphocytes were identified as events with PreCP fluorescence above 103 on logarithmic scale). (S3) CD4-positive cells (T helper lymphocytes were identified as events with APCCy7 fluorescence above 102 on logarithmic scale) and CD8-positive cells (T cytotoxic lymphocytes were identified as events with APC fluorescence above 103 on logarithmic scale). IFN-γpos cells were identified as cells positive for PeCy7 above 103, respectively, from gate CD3 (S4a), CD4 (S5a), and CD8 (S6a). TNF-αpos cells were identified as cells positive for PeCy7 above 103, respectively, from gate CD3 (S7a), CD4 (S8a), and CD8 (S9a). FMO controls are represented on S4, S5, S6, S7, and S8 histograms. [file 8478750.f1.pdf]

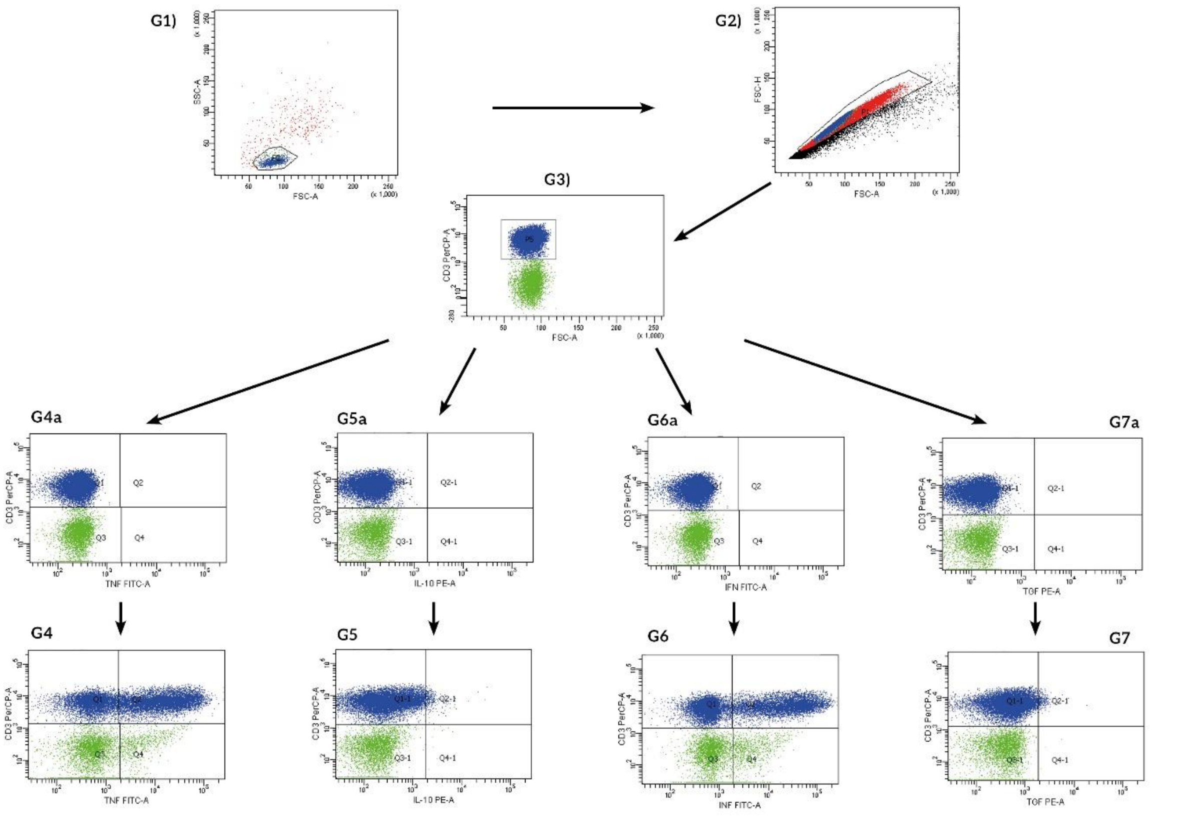

Supplementary data to figure 1: Lymphocytes after PMA stimulation. Gating strategy: lymphocytes were identified on FSC/SSC dot plot G1) as cells within 50-100 x 10^3 on FSC and below 50 x 10^3 on SSC. G2) doublets exclusion. G3) CD3 positive cells (T lymphocytes were identified as events with PreCP fluorescence above 10^3 on logarithmic scale G4) TNF - α positive T lymphocytes were identified as $CD3^+$ lymphocytes from G3 gate with fluorescence higher than fluorescence minus one from G4a. G5) IL-10 positive T lymphocytes were identified as $CD3^+$ lymphocytes from G3 gate with fluorescence higher than fluorescence minus one from G5a. G6) INF – γ positive T lymphocytes were identified as $CD3^+$ lymphocytes from G3 gate with fluorescence higher than fluorescence minus one from G6a. G7) TGF - β positive T lymphocytes were identified as $CD3^+$ lymphocytes from G3 gate with fluorescence higher than fluorescence minus one from G7a. FMO controls are represented on G4a, G5a, G6a, Ga7, G8a dot plots.

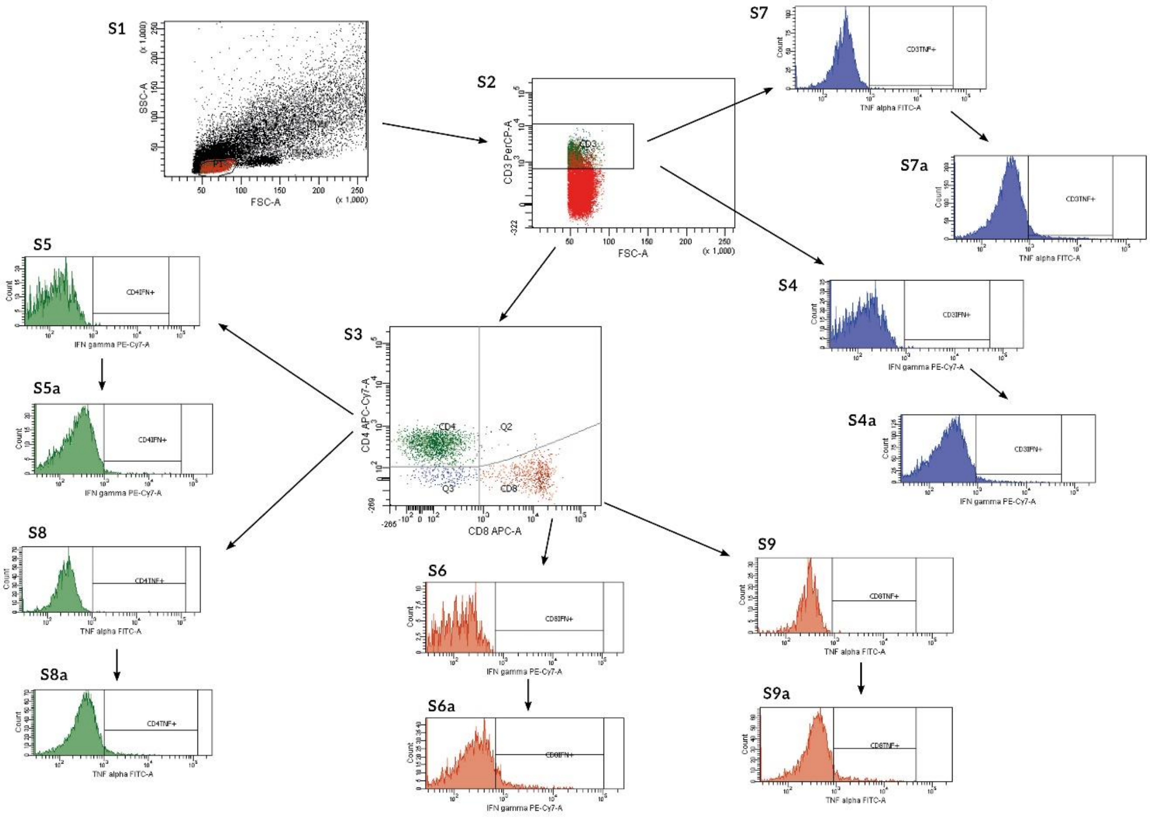

Supplementary data to figure 2: Lymphocytes after PHA stimulation. Gating strategy: lymphocytes were identified on FSC/SSC dot plot S1) as cells within 50-100 x 10^3 on FSC and below 50 x 10^3 on SSC, S2) CD3 positive cells (T lymphocytes were identified as events with PreCP fluorescence above 10^3 on logarithmic scale S3) CD4 positive cells (T helper lymphocytes were identified as events with APC-Cy7 fluorescence above 10^2 on logarithmic scale and CD8 positive cells (T cytotoxic lymphocytes were identified as events with APC fluorescence above 10^3 on logarithmic scale. IFN – γ ^{pos} cells were identified as cells positive for PeCy7 above 10^3 respectively from gate CD3 (S4a), CD4(S5a), CD8(S6a). TNF -α ^{pos} cells were identified as cells positive for PeCy7 above 10^3 respectively from gate CD3(S7a), CD4(S8a), CD8(S9a). FMO controls are represented on S4, S5, S6, S7, S8 histograms.
